# Ginsenoside Rh2 Inhibits NLRP3 Inflammasome Activation and Improves Exosomes to Alleviate Hypoxia-Induced Myocardial Injury

**DOI:** 10.3389/fimmu.2022.883946

**Published:** 2022-07-05

**Authors:** Zhongwen Qi, Zhipeng Yan, Yueyao Wang, Nan Ji, Xiaoya Yang, Ao Zhang, Meng Li, Fengqin Xu, Junping Zhang

**Affiliations:** ^1^ Postdoctoral Research Station of China Academy of Chinese Medical Sciences, Institute of Gerontology, Xiyuan Hospital, China Academy of Chinese Medical Sciences, Beijing, China; ^2^ Medical Experiment Center, First Teaching Hospital of Tianjin University of Traditional Chinese Medicine, Tianjin, China; ^3^ National Clinical Research Center for Chinese Medicine Acupuncture and Moxibustion, First Teaching Hospital of Tianjin University of Traditional Chinese Medicine, Tianjin, China; ^4^ Graduate School, Tianjin University of Traditional Chinese Medicine, Tianjin, China; ^5^ Institute of Hypertension, Jiangsu Province Hospital of Chinese Medicine, Affiliated Hospital of Nanjing University of Chinese Medicine, Nanjing, China

**Keywords:** hypoxia and inflammation, cardiomyocyte, ginsenoside, exosomes, myocardial injury

## Abstract

The inflammatory microenvironment after acute myocardial infarction (MI) is a key limiting factor in the clinical application of stem cell transplantation and paracrine exosome therapy. Qishen Yiqi Pills contain a saponin ingredient called Ginsenoside Rh2 (Rh2) which exhibits a certain therapeutic effect on MI. However, the mechanism by which Rh2 alleviates the inflammatory microenvironment and improves the therapeutic efficiency of exosomes remains enigmatic. Here, we found that Rh2 attenuated the adverse effect of oxygen-glucose deprivation (OGD)-induced cellular injury, an *in vitro* pathological model of MI. Confocal microscopy revealed that DiI-labeled BMSCs-derived exosomes exhibited an increased homing ability of cardiomyocytes, which, in turn, inhibited the nuclear translocation of NF-κB p65 and NLRP3 inflammasome activation, thereby alleviating the inflammatory microenvironment and further facilitating the homing of exosomes to cardiomyocytes by forming a feed-forward enhancement loop. Additionally, we found that Rh2 could regulate the HMGB1/NF-κB signaling pathway to improve the OGD environment of cardiomyocytes, increasing the efficiency of the feed-forward loop. In conclusion, we found that Rh2 can improve the inflammatory microenvironment by enhancing the protection of exosomes against myocardial injury, providing new insights into the indirect modification of exosomes by Rh2 in MI treatment.

## Introduction

Cardiovascular diseases, including acute myocardial infarction (MI) are among the most deadly diseases worldwide, causing great morbidity and mortality annually (1). The strong inflammatory response accompanied by the massive death of myocardial cells after MI results in progressive deterioration of cardiac function and even heart failure (2). Stem cell therapy has emerged as a promising strategy for preventing myocardial cell loss, thereby promoting functional recovery after MI (3, 4). Being multipotent stem cells, bone marrow mesenchymal stem cells (BMSCs) are ideal candidates for stem cell therapy because of their relatively low immunogenicity (5). However, animal MI models and clinical trials have shown that the survival of transplanted stem cells is low, which remains a technical bottleneck for the clinical application of stem cell therapies (6). Transplanted stem cells play a cardioprotective role mainly through paracrine exosomes that are involved in anti-inflammation, anti-apoptosis, and angiogenesis promotion (7, 8). However, the inflammatory response after MI decreases the retention and survival of exosomes, which greatly undermines the therapeutic effect of exosomes (9, 10). Hence, it is urgent to find a solution to relieve the inflammatory microenvironment after MI.

Various methods have been developed to improve the therapeutic efficacy of exosomes, including drug pretreatment (11), hypoxia preadaptation (12), gene editing (13), and hydrogel tissue engineering (14, 15). However, these methods have not been proven to be applicable in clinics. Traditional Chinese Medicine (TCM) holds important clinical potential in drug pretreatment. Qishen Yiqi Pills (QSYQ) were approved by the China Food and Drug Administration (CFDA) in 2003 (Approval number of CFDA: Z20030139) (14), which is composed of *Hedysarum multijugum Maxim* (HM), *Radix salviae* (SM), *Panax notoginseng* (PN), and *Dalbergia odorifera* (DO) at a proportion of 10:5:1:0.067 (16). Due to its significant clinical efficacy and safety, QSYQ is widely used in patients with cardiovascular disease, Qi deficiency, and blood stasis syndrome (17). In our previous work, which combined network pharmacology and molecular docking, we clarified that Ginsenoside Rh2 (Rh2) is an active component of QSYQ and has a protective effect on cardiomyocytes under the condition of oxygen–glucose deprivation (OGD). Meanwhile, Rh2 has a good binding site with HMGB1, and under the condition of OGD, Rh2 decreases the expression of HMGB1 (18). Moreover, Rh2 was found to improve cardiac function and exert cardioprotective effects by scavenging tissue ROS, increasing SOD expression, and reducing oxidative stress in a diabetic cardiomyopathy rat model (19). Furthermore, it has been observed that Rh2 can regulate immune function and improve myocardial ischemia through its anti-allergic activity (20). However, whether Rh2 can improve the inflammatory microenvironment and enhance the function of exosomes in MI is unknown.

NLRP3 inflammasomes have garnered great research interest as a core part of inflammatory machinery. It is a macromolecular multi-protein complex recognizing pathogen-associated molecular patterns (PAMPs) or damage-associated molecular patterns (DAMPs) through pattern recognition receptors (PRRs) (21). Upon activation, the NLRP3 inflammasome releases an array of inflammatory factors causing myocardial injury, including interleukin-1β (IL-1β) and tumor necrosis factor α (TNF-α) (22, 23). Toll-like receptor 4 (TLR4) mediates the inflammatory response in the myocardium, aggravating myocardial injury. High mobility group box 1 (HMGB1) is an endogenous ligand of TLR4. Once TLR4/HMGB1 is activated, the signal can be further transduced through myeloid differentiation factor 88-dependent and independent pathways to activate NF-κB (24), which further activates the NLRP3 inflammasome through DAMPs (25), promoting the release of downstream inflammatory factors and oxygen-free radicals. Therefore, the HMGB1/NF-κB signaling pathway is essential for initiating inflammatory responses (26).

In this study, we investigated the therapeutic effect of Rh2 on OGD injury and elucidated the pertinent mechanism, providing a new perspective for designing an efficient treatment of MI. Our results suggest that Rh2 can block NF-κB nuclear translocation and inhibit NLRP3 inflammasome activation, which improves the homing of the exosome to cardiomyocytes, thereby providing protection against MI.

## Materials and Methods

### BMSC Isolation and Culture

All animal experiments were approved by the Animal Ethics Committee of Tianjin University of TCM (approval number: TCM-LAEC2018032). Male specific-pathogen-free (SPF) Sprague–Dawley (SD) rats (5–6 weeks old, 200 ± 20 g) were purchased from Huafukang Biotechnology Co., Ltd. (Beijing, China). The animals were housed in a clean-grade animal room of the Experimental Center of Nankai Hospital, Tianjin, China. The room conditions were maintained at 22–24 °C, 40–60% humidity, and 12 h light/dark cycles. The animals were fed free diets for 3 days. According to experimental methods provided in the literature (27), primary BMSCs were extracted. When grown to 80% confluence, the cells were digested with trypsin for 90 s and then transferred to a new culture dish. After 3–4 generations, cells are collected and used for experiments.

### Identification of BMSCs

The collected cells were stained with antibodies against CD45, CD31, CD44, and CD29 for 30 min at 4°C in the dark. Then, after two washes with 1× phosphate-buffered saline (PBS), the cells were resuspended in 500 μl of 1× PBS. Further, the cells were sorted on a FACS AriaIII flow cytometer (BD Immunocytometry Systems) and analyzed using the FlowJo software (Ashland, OR).

### Exosomes Isolation, Identification, and Labeling

Exosomes were isolated from a BMSC-conditioned medium by differential centrifugation (28). Then, the exosomes were resuspended in 1× PBS and stored at −80°C for use. The shape and size of exosomes were determined by transmission electron microscopy (TEM, FEI, Tecnai G2 Spirit BioTwin, USA) and nanoparticle tracking analysis (NTA, PARTICLE METRIX, ZetaVIEW S/N 17-310, Germany). Besides, exosome-specific marker proteins, including CD9, CD63, and TSG101, were identified by immunoblotting (Abcam, USA). To further assess the uptake and distribution of purified exosomes in cardiomyocytes at different time points, exosomes were labeled using a DiI Fluorescent Cell Kit (Beyotime, Shanghai, China) following the instructions of the manufacturer.

### H9c2 Cell Culture, OGD Model Construction, and Rh2 Treatment

H9c2 cells derived from embryonic rats were purchased from the Prosper Life Technology Co., Ltd. (Wuhan, China) and cultured in 10% fetal bovine serum (FBS) (Invitrogen, Carlsbad, CA, USA) and 1% streptomycin (Sigma, San Francisco, USA) at 37°C in a humidified 5% CO_2_–95% air incubator under standard conditions. The log-phase H9c2 cells were collected and plated at a density of 70% of the calculated cell density on the next day. The next day, the medium was replaced with a serum-free and sugar-free DMEM, and the cells were cultured for 12 h in a hypoxic chamber with 1% oxygen.

For Rh2 treatment, Rh2 powders (C_36_H_62_O_8_, molecular weight: 622.87, purity>98%) (Med Chem Express Co.) were dissolved in dimethyl sulfoxide (DMSO) at a concentration of 50 mM as a stock solution. According to our previous findings, Rh2 at 2 µM can effectively inhibit H9c2 cell death (18). H9c2 cells were divided into the following groups: Control group, Model group, Rh2 group (2 µM), Exo group (10 µg/ml), and Rh2 + exo group (2 µM combined with 10 µg/ml exosome).

### Immunofluorescence Staining and Confocal Microscopy

Cells in each group were mounted onto cell slides and then incubated with primary antibodies overnight at 4°C. On the next day, following three washes with 1× PBS, the slides were incubated with Alexa Fluor 488 goat anti-rabbit antibody (YEASEN, 1:200, China) or Alexa Fluor 594 goat anti-mouse antibody (YEASEN, 1:200, China) for 1 h at room temperature. After that, the slides were washed with PBS three times and stained with DAPI. The cells were observed under a laser scanning confocal microscope (NIKON, Japan) for observation. The primary antibodies used in this study were as follows: ASC (Novusbio, 1:100, USA) and Cryopyrin (HuaBio, 1:100, China) for staining major components of NLRP3; NF-κB p65 (Abcam, 1:100, USA) against NF-κB.

### H9c2 Cells Nuclear and Cytoplasmic Protein Extraction

Following the instructions of the manufacturer of a cytoplasmic and nucleus extraction kit (Beyotime, Shanghai, China), H9c2 cells were treated with a recommended number of cytoplasmic extraction reagents. Then, the supernatant containing H9c2 cytoplasmic proteins was collected. Subsequently, 50 µl of the nuclear protein extraction reagents with the protease inhibitor PMSF (final concentration: 1 mM) (Beyotime, Shanghai, China) were added to the pellet. Following the centrifugation, the supernatant containing H9c2 nuclear proteins was collected.

### Western Blot Analysis

Exosomes and H9c2 cells were lysed with RIPA buffer containing protease inhibitors (Sangon Biotech, China) on ice. The crude lysate was centrifuged and the supernatant was collected. The protein concentration was determined using a BCA protein assay kit (PIELS). Protein lysates were separated on 10% SDS-PAGE and blotted onto polyvinylidene fluoride membranes (Millipore, USA). The membrane was blocked with 5% skim milk in TBST at room temperature and incubated with the primary antibodies, including CD63 (1:1,000, Abcam, USA), TSG101 (1:1,000, Abcam, USA), NF-κB p65 (1:100, Abcam, USA), HMGB1 (1:1,000, HuaBio, China), and NLRP3 (1:1,000, HuaBio, China) overnight at 4°C. The next day, the membrane was washed with 1× PBS, followed by incubation with HRP-conjugated secondary antibodies at room temperature for 1 h. The protein bands were visualized with chemiluminescence reagents and were observed on a multifunctional chemiluminescence imager (CLiNX, 620028-08Q, China). The gray values were analyzed by the ImageJ software. The experiment was repeated at least three times.

### Statistical Analysis

Statistical analyses were performed using the GraphPad Prism 8.0 software (GraphPad Software, San Diego, CA, USA). A Student’s *t*-test was used for statistical comparisons between two groups, and a one-way ANOVA test was employed for multi-group comparisons. All the experiments were performed at least three times, and the data were plotted as mean ± SD. *P <*0.05 was considered statistically significant.

## Results

### Characterization of BMSCs and Identification of Exosomes Derived From BMSCs

**Figure 1:**
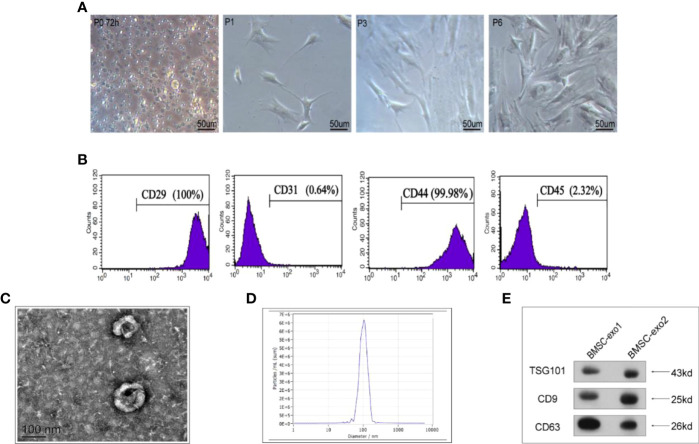
Characterization of BMSCs and identification of exosomes derived from BMSCs. **(A)** Morphology of BMSCs of different generations under the microscope. **(B)** Flow cytometry of the cell surface markers of BMSCs (CD29, CD31, CD44, and CD45). **(C)** Transmission electron microscopy was used to identify the shape of exosomes. **(D)** Nanoparticle tracking analysis was utilized to analyze the particle size of exosomes. **(E)** Detection of exosomal protein markers was performed using western blotting (TSG101, CD9, and CD63).

To pinpoint the right time for exosome extraction, we observed the state of BMSCs each day during cell culture. At 24 h post-inoculation, only a few cells were adherent; after 48 h, a large fraction of BMSCs became adherent with different shapes, albeit most of them being round; after 72 h, the cell shape became homogenous and almost all cells were adherent (**Figure 1A**). Accordingly, we determined that the third generation of BMSCs are typical adherent spindle cells. Moreover, flow cytometry showed that these cells were CD29 and CD44 positive, with the positive rates being 100 and 99.98%, respectively, but CD45 and CD31 negative, with the positive rates being merely 2.32 and 0.64%, respectively (**Figure 1B**).

Next, we used transmission electron microscopy and a nanoparticle tracing system to analyze the shape and size of BMSC-derived exosomes. We observed that, in general, they were round or oval bilayer lipid vesicles with a distinct membrane structure and an average size of 112.8 nm (**Figures 1C, D**). Furthermore, western blotting showed that BMSCs-derived exosomes expressed exosome-specific proteins, including CD9, CD63, and TSG101 (**Figure 1E**).

### Different Components of NLRP3 Inflammasome Are Activated Under OGD Induction in H9c2 Cells

**Figure 2:**
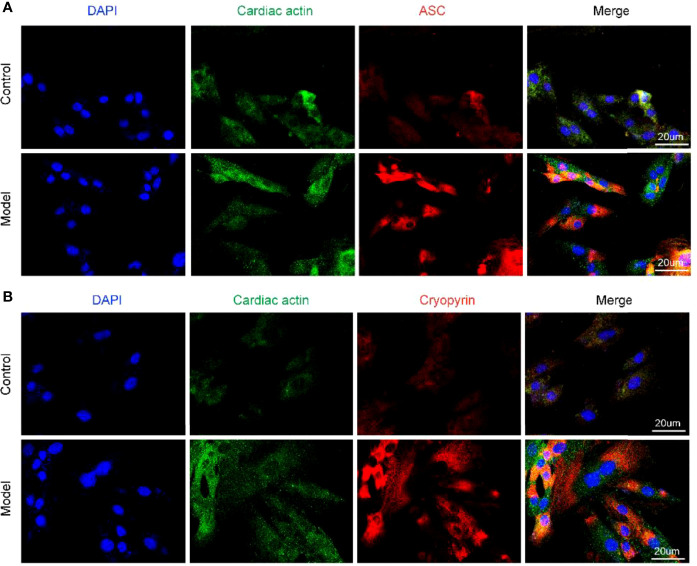
Different components of NLRP3 inflammasome are activated under OGD induction in H9c2 cells. **(A)** The protein expression levels of the ASC (red color) in H9c2 by immunofluorescence, and the slides were counterstained with DAPI (blue). **(B)** The protein expression levels of the Cryopyrin (red color) in H9c2 by immunofluorescence, and the slides were counterstained with DAPI (blue).

We previously observed that the OGD model of hypoxia, after culture for 12 h, exhibited cell membrane damage, rupture, and swelling. However, whether this process triggers the activation of NLRP3 inflammasomes is unclear. To address this, we examined the NLRP3 inflammasome activation state under OGD induction by immunofluorescence. After 12 h of OGD stimulation, the fluorescence intensities of ASC and NLRP3 (Cryopyrin) were significantly enhanced (**Figures 2A, B**), suggesting that OGD can activate the NLRP3 inflammasomes in H9c2 cells, which may further aggravate the cell damage.

### Nuclear Translocation of NF-κB p65 Occurred Under OGD Induction in H9c2 Cells

**Figure 3:**
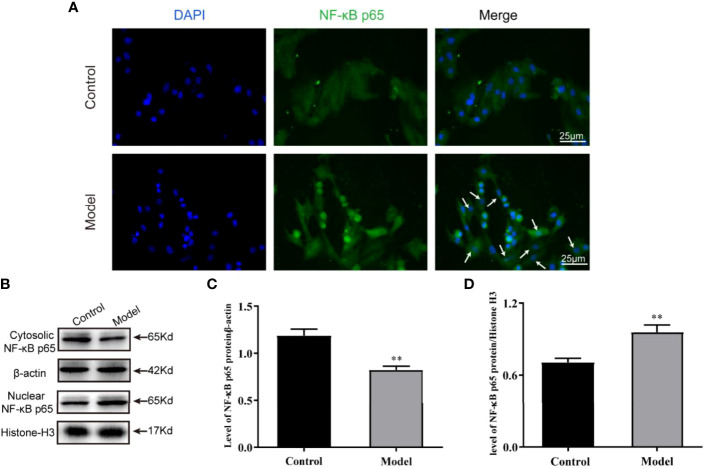
Nuclear translocation of NF-κB p65 occurred under OGD induction in H9c2 cells. **(A)** The protein expression levels of the NF-κB p65 (green color) in cardiomyocyte nuclear by immunofluorescence, and the slides were counterstained with DAPI (blue). Representative images of western blotting **(B)** and quantitative data **(C, D)** of the protein levels of NF-κB p65 in the nuclear and cytosolic. One-way ANOVA test was used for statistical analyses. Bars represent means ± SD; **P <0.01. ns, no significance.

The activation of NLRP3 inflammasomes is mediated by DAMPs and is regulated by the microenvironment of the cardiomyocytes. As a key immune factor, NF-κB activates the inflammatory cascade represented by the NLRP3 inflammasome and accelerates cell damage when nuclear translocation occurs. To clarify whether the nuclear translocation of NF-κB p65 occurs in the cardiomyocytes of the OGD model, we used immunofluorescence labeling and Western blot to detect the abundance of NF-κB p65 in the nucleus and cytoplasm. We found that NF-κB p65 was indeed translocated to the nucleus after 12 h of stimulation by OGD, resulting in activation of the NLRP3 inflammasome (**Figures 3A–D**).

### Rh2 Promoted Exosome Inhibition of NF-κB Nuclear Translocation

**Figure 4:**
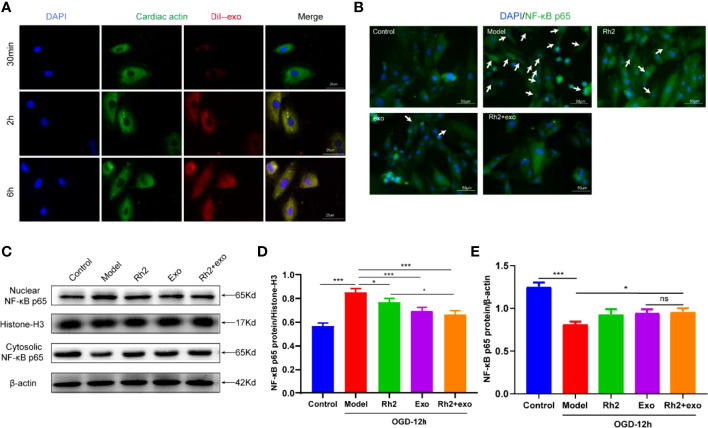
Ginsenoside Rh2 promoted exosome inhibition of NF-κB nuclear translocation. **(A)** Enrichment of DiI-labeled exosomes in cardiomyocytes at different time points (30 min/2 h/6 h). **(B)** The protein expression levels of the NF-κB p65 (green color) in cardiomyocyte nuclear by immunofluorescence, and the slides were counterstained with DAPI (blue). Representative images of western blotting **(C)** and quantitative data **(D, E)** of the protein levels of NF-κB p65 in the nuclear and cytosolic. One-way ANOVA test was used for statistical analyses. Bars represent means ± SD; *P < 0.05, ***P < 0.001. ns, no significance.

Considering the association between the translocation of NF-κB and exosomes, we speculated that Rh2 may inhibit the nuclear translocation of NF-κB to increase the retention of exosomes in injured cardiomyocytes, thereby protecting cardiac functions. To test this, we first treated cardiomyocytes with DiI-labeled exosomes and observed the enrichment of exosomes in cardiomyocytes at different time points (30 min/2 h/6 h). The exosomes homed to the surrounding cardiomyocytes and enriched them with the prolongation of observation time (**Figure 4A**).

The homing activity of exosomes is affected by the inflammatory microenvironment under OGD conditions. To explore whether Rh2 could improve the harsh microenvironment to promote the recruitment of exosomes to cardiomyocytes, we treated the OGD model with Rh2, Exo, and Rh2 + exo. All treatments decreased NF-κB nuclear translocation compared with the control group. In particular, the Rh2 + exo group showed the strongest capacity to increase the NF-κB p65 duration in the nucleus (**Figures 4B–E**). In conclusion, Rh2 can improve the cardiomyocyte microenvironment and enhance the therapeutic effect of exosomes by inhibiting the nuclear translocation of NF-κB.

### Rh2 Enhanced Exosome Inhibition of Different Components of NLRP3 Inflammasome Activation

**Figure 5:**
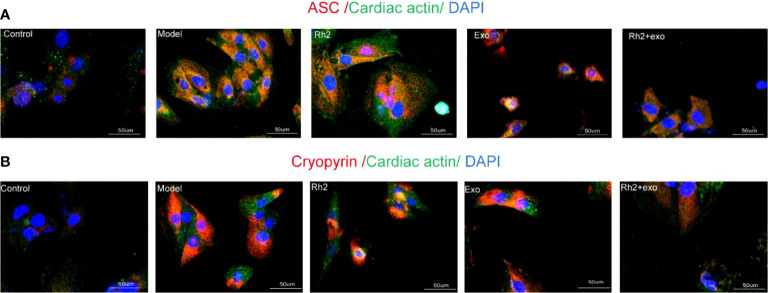
Ginsenoside Rh2 enhanced exosome inhibition of different components of NLRP3 inflammasome activation. **(A, B)** The protein expression levels of the ASC (red color) and Cryopyrin (red color) in H9c2 by immunofluorescence, and the slides were counterstained with DAPI (blue).

Since Rh2 can reduce the level of NF-κB in myocardial cytoplasm, we further evaluated its effect on NLRP3 inflammasome activation. The activation of NLRP3 inflammasomes in the Rh2 + exo group was lower than that in the Rh2 group and the exosome group (**Figures 5A, B**). These results suggest that Rh2 can inhibit NLRP3 inflammasome activation, possibly by enhancing the homing of exosomes to cardiomyocytes, reducing the nuclear translocation of NF-κB, and suppressing the inflammatory cascade.

### Rh2 Inhibited NLRP3 Inflammasome Activation *via* HMGB1/NF-κB Signaling to Promote Exosome Therapy for Myocardial Injury

**Figure 6:**
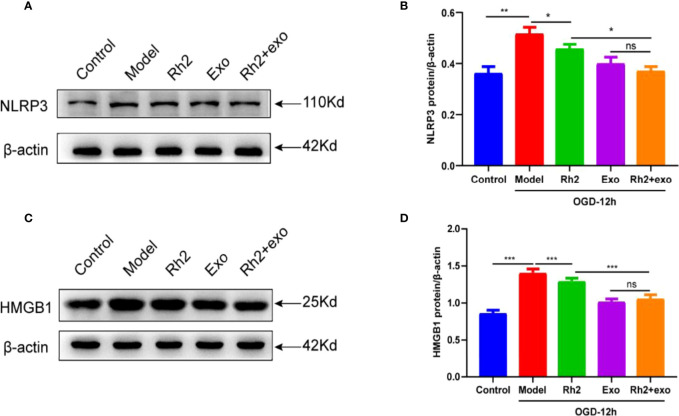
Ginsenoside Rh2 inhibited NLRP3 inflammasome activation *via* HMGB1/NF-κB signaling to promote exosome therapy for myocardial injury. Representative images of western blotting **(A, C)** and quantitative data **(B, D)** of the protein levels of NLRP3 and HMGB1. One-way ANOVA test was used for statistical analyses. Bars represent means ± SD; *P < 0.05, **P < 0.01, ***P < 0.001. ns, no significance.

To further elucidate the mechanism underlying how Rh2 enhances the cardioprotective effect of exosomes, we examined the expression level of HMGB1. HMGB1 is implicated in a series of inflammatory responses and plays an important role in the nuclear translocation of NF-κB. In addition to activating downstream NF-κB, the NLRP3 inflammasome can accelerate the release of HMGB1 from the nucleus, resulting in a feed-forward loop driving inflammatory responses. As such, HMGB1/NF-κB may play an important role in the activation of the NLRP3 inflammasome. To check this possibility, we treated the OGD models and found that the addition of Rh2 + exo significantly reduces the protein level of NLRP3 (**Figures 6A, B**). We also detected similar effects on HMGB1 protein (**Figures 6C, D**). These results clarify that Rh2 can inhibit NLRP3 inflammasome activation through the HMGB1/NF-κB pathway.

## Discussion

Recently, stem cell therapy involving paracrine exosomes has become a promising strategy for treating cardiovascular diseases. However, the inflammatory response after MI impairs stem cell migration, dramatically reducing the therapeutic effect of exosomes (29). Therefore, it is imperative to find a solution that can effectively improve the inflammatory microenvironment after MI. To this end, we explored whether Rh2, an active ingredient of TCM, could improve the harsh inflammatory microenvironment to enhance the therapeutic effect of exosomes. The main findings are as follows: (1) NLRP3 inflammasome was activated under the OGD condition in cardiomyocytes; (2) After 12 h of the OGD condition, NF-κB p65 translocation from the nucleus to the cytoplasm appeared in cardiomyocytes; (3) DiI-labeled BMSCs-derived exosomes exhibited an increased homing ability of cardiomyocytes; (4) Rh2 reduced the activation of NLRP3 inflammasome through the HMGB1/NF-κB signaling pathway, thereby attenuating the activation of the NLRP3 inflammasome, improving the inflammatory microenvironment and increasing the enrichment of exosomes in cardiomyocytes.

Exosomes have emerged as a promising therapy for promoting cardiac repair (30), with anti-apoptotic (31), anti-inflammatory (32), and pro-angiogenic activities (33). These effects have important clinical significance for improving cardiac function. However, the harsh microenvironments, such as calcium overload, acidosis, and reactive oxygen species production in the injured myocardium, significantly dampen the survival rate of the transplanted exosomes and undermine the incidence of homing to the injured tissue, posing great limits on their clinical application as cell-free therapies (34). The injection of exosomes into myocardial tissue at the early stage of MI can effectively reduce inflammation, inhibit apoptosis, and increase SDF-1 expression (35). Additionally, pre-hypoxic treatment of stem cell-derived exosomes in cardiomyocytes can significantly promote angiogenesis, reduce cardiac fibrosis, and improve cardiac function (36). Therefore, exosomes have been widely considered as a new therapeutic vector for cardiac repair.

To improve the therapeutic efficacy of exosomes, various protocols have been developed over the last decade, including drug preconditioning (11), hypoxia preconditioning (12), gene editing (13), and hydrogel tissue engineering (14, 15). TCM holds an important clinical potential in drug pretreatment. For example, Tongxinluo Capsule is effective for treating coronary heart disease in clinical practice, preventing exosome damage under hypoxic conditions by preconditioning BMSCs (37). Therefore, the development of clinically effective drugs as boosters for cell-free therapy will be beneficial.

Under the guidance of the integration of TCM network pharmacology and molecular docking technology, we conducted a bioinformatics analysis of QSYQ and found that Rh2 could be an effective ingredient for protecting myocardial cells under OGD conditions (18). The NLRP3 inflammasome is an important inflammatory module and triggers immune responses by recognizing different damaging molecules, posing great limits on cell therapies (38). However, it is still unclear whether Rh2 can enhance the effect of BMSC-derived exosomes on MI treatment. To test this hypothesis, we established an *in vitro* OGD model to mimic MI. In this model, although BMSC-derived exosomes showed a cardiomyocyte homing trend, a large fraction of exosomes were enriched around cardiomyocytes even after 6 h of hypoxia. Additionally, NLRP3 inflammasomes were enhanced to varying degrees, and NF-κB p65 was translocated from the nucleus to the cytoplasm. After treatment with three different regimens of exosomes, Rh2 alone or Rh2 combined with exosomes effectively reduced the expression of ASC, an active component of the NLRP3 inflammasome, and inhibited the nuclear translocation of NF-κB p65, and Rh2 combined with exosomes showed a more significant effect. These results indicate that Rh2 can attenuate the activation of the NLRP3 inflammasomes by inhibiting the nuclear translocation of NF-κB p65, thereby improving the inflammatory microenvironment and increasing the enrichment of exosomes into cardiomyocytes.

It has been reported that NF-κB nuclear translocation is affected by HMGB1 (39). HMGB1 is released by necrotic and apoptotic cells (40, 41), and is a key protein in the progression of various inflammatory diseases (42). NLRP3 inflammasomes can facilitate the release of HMGB1 from the nucleus to promote ischemia-related inflammatory responses (43), which activates the downstream inflammatory proteins, such as MAPK, IRF, and NF-κB (44). HMGB1 is up-regulated in a myocardial ischemia–reperfusion injury model, which increases the expression level of phosphorylated NF-κB p65, activates the NLRP3 inflammasome and induces the release of mature IL-1β (45). In this study, we found that HMGB1 was significantly upregulated in the cardiomyocyte OGD model, which was reversed by the application of Rh2 alone or Rh2 + exo. These results demonstrate that Rh2 reduces the activity of the NLRP3 inflammasome, probably through the HMGB1/NF-κB signaling pathway.

**Figure 7:**
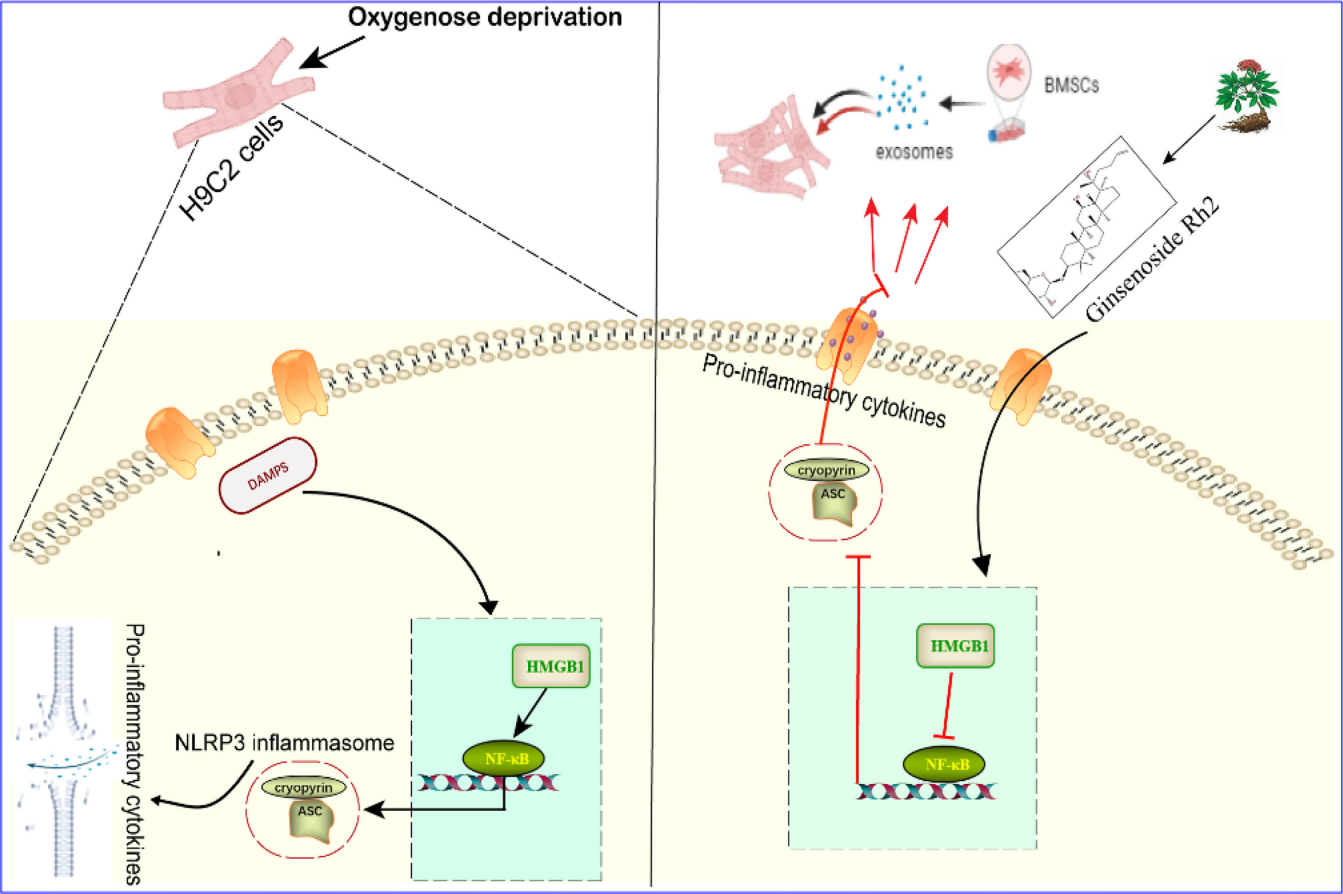
Effect and mechanism of Ginsenoside Rh2 promoted BMSC-derived exosomes in the treatment of cardiomyocyte injury under oxygen glucose deprivation conditions.

In conclusion, we have studied the therapeutic effect of Rh2 combined with exosomes on OGD-induced injury in myocardial cells, providing a possible model whereby Rh2 blocks NF-κB nuclear translocation through the HMGB1/NF-κB signaling pathway, thereby inhibiting the activation of the NLRP3 inflammasome and improving the homing to cardiomyocytes of the exosome, which ultimately confers protection to cardiomyocytes. Taken together, these findings suggest that Rh2 can improve the inflammatory microenvironment by enhancing the protection of exosomes against myocardial injury (**Figure 7**). Although large-scale animal and clinical trials are required to provide new insights into the indirect modification of exosomes by Rh2 in MI treatment.

## Data Availability Statement

The datasets presented in this study can be found in online repositories. The names of the repository/repositories and accession number(s) can be found in the article/supplementary material.

## Author Contributions

ZQ was in charge of doing experimental design, basic experiments (including index detection and pathological analysis), data statistics, and writing articles. ZY, YW, and NJ were in charge of doing the basic experiments, including index detection and pathological analysis. JZ was in charge of directing the experimental work and reviewing this article. XY and AZ were in charge of doing the network pharmacology and molecular docking analysis. ML and FX revised the manuscript. All authors contributed to the article and approved the submitted version.

## Funding

This study was supported by the National Natural Science Foundation of China (Grant Nos. 81804046, 81774232) and Chinese Medicine Inheritance and Innovation Talent Project (QI Huang Scholars) (No.203).

## Conflict of Interest

The authors declare that the research was conducted in the absence of any commercial or financial relationships that could be construed as a potential conflict of interest.

## Publisher’s Note

All claims expressed in this article are solely those of the authors and do not necessarily represent those of their affiliated organizations, or those of the publisher, the editors and the reviewers. Any product that may be evaluated in this article, or claim that may be made by its manufacturer, is not guaranteed or endorsed by the publisher.
